# Supplementary material for: From sequence to enzyme mechanism using multi-label machine learning
Source: BMC Bioinformatics. 2014 May 19;15:150. doi: 10.1186/1471-2105-15-150 (PMC4229970; doi:10.1186/1471-2105-15-150)
Supplement: Additional file 2 — Java code of ml2db. Additional file ml2db_code.tar.gz contains the Java source code to run the multi-label machine learning experiments and save the results to database. The code’s Javadoc is included. [file 1471-2105-15-150-S2.zip › additional file 2/ml2db/ml2db/doc/test/dataharness/DataTwo.html]

DataTwo


---


|  |  |  |
| --- | --- | --- |
| **Overview** \| **Package** \| **Class** \| **Use** \| **Tree** \| **Deprecated** \| **Index** \| **Help** | |  |
| **PREV CLASS**   **NEXT CLASS** | **FRAMES**    **NO FRAMES**     **All Classes** |  |
| SUMMARY: NESTED \| FIELD \| CONSTR \| METHOD | DETAIL: FIELD \| CONSTR \| METHOD |  |


---


## test.dataharness Class DataTwo

```
java.lang.Object
  test.dataharness.DataTwo
```

---

``` public class DataTwo extends java.lang.Object ```

Class

**Version:**
:   25 Feb 2011

**Author:**
:   Luna De Ferrari luna.deferrari-at-ed.ac.uk

---

| **Field Summary** | |
| --- | --- |
| `static java.lang.String` | `arffEmptyInstance` |
| `static java.lang.String` | `arffInstance1` |
| `static java.lang.String` | `arffInstance1b` |
| `static java.lang.String` | `arffInstance2` |
| `static java.lang.String` | `arffInstance4` |
| `static java.lang.String` | `arffInstanceAttVoid` |
| `static java.lang.String` | `arffInstanceClassVoid` |
| `static java.lang.String` | `DATASET_NAME` |
| `static java.lang.String` | `INST1` |
| `static java.lang.String` | `INST2` |
| `static java.lang.String` | `INST3` |
| `static java.lang.String` | `INST4` |
| `static int` | `NUMBER_OF_INSTANCES_IN_TEST_ARFF` |


**Constructor Summary**

`DataTwo()`


| **Method Summary** | |
| --- | --- |
| `static uk.ac.ed.inf.utils.maputils.OneToManyMap<java.lang.String,java.lang.String>` | `getInstanceAttributeMap()` |
| `static uk.ac.ed.inf.utils.maputils.OneToManyMap<java.lang.String,java.lang.String>` | `getInstanceClassMap()` |

**Methods inherited from class java.lang.Object**

`equals, getClass, hashCode, notify, notifyAll, toString, wait, wait, wait`

**Field Detail**

### NUMBER\_OF\_INSTANCES\_IN\_TEST\_ARFF

```
public static int NUMBER_OF_INSTANCES_IN_TEST_ARFF
```

---


### arffInstance1

```
public static final java.lang.String arffInstance1
```

**See Also:**: Constant Field Values

---


### arffInstance1b

```
public static final java.lang.String arffInstance1b
```

**See Also:**: Constant Field Values

---


### arffInstance2

```
public static final java.lang.String arffInstance2
```

**See Also:**: Constant Field Values

---


### arffInstance4

```
public static final java.lang.String arffInstance4
```

**See Also:**: Constant Field Values

---


### arffEmptyInstance

```
public static final java.lang.String arffEmptyInstance
```

**See Also:**: Constant Field Values

---


### arffInstanceClassVoid

```
public static final java.lang.String arffInstanceClassVoid
```

**See Also:**: Constant Field Values

---


### arffInstanceAttVoid

```
public static final java.lang.String arffInstanceAttVoid
```

**See Also:**: Constant Field Values

---


### INST1

```
public static final java.lang.String INST1
```

**See Also:**: Constant Field Values

---


### INST2

```
public static final java.lang.String INST2
```

**See Also:**: Constant Field Values

---


### INST3

```
public static final java.lang.String INST3
```

**See Also:**: Constant Field Values

---


### INST4

```
public static final java.lang.String INST4
```

**See Also:**: Constant Field Values

---


### DATASET\_NAME

```
public static final java.lang.String DATASET_NAME
```

**See Also:**: Constant Field Values


**Constructor Detail**

### DataTwo

```
public DataTwo()
```


**Method Detail**

### getInstanceAttributeMap

```
public static uk.ac.ed.inf.utils.maputils.OneToManyMap<java.lang.String,java.lang.String> getInstanceAttributeMap()
```

---


### getInstanceClassMap

```
public static uk.ac.ed.inf.utils.maputils.OneToManyMap<java.lang.String,java.lang.String> getInstanceClassMap()
```


---


|  |  |  |
| --- | --- | --- |
| **Overview** \| **Package** \| **Class** \| **Use** \| **Tree** \| **Deprecated** \| **Index** \| **Help** | |  |
| **PREV CLASS**   **NEXT CLASS** | **FRAMES**    **NO FRAMES**     **All Classes** |  |
| SUMMARY: NESTED \| FIELD \| CONSTR \| METHOD | DETAIL: FIELD \| CONSTR \| METHOD |  |


---
